# HMGB1 Upregulates MMP-1-Mediated Mesothelial–Mesenchymal Transition and Promotes Pleural Fibrosis in Tuberculous Pleural Effusion

**DOI:** 10.3390/ijms27135961

**Published:** 2026-07-02

**Authors:** Wei-Lin Chen, Kai-Ling Lee, Mei-Chuan Chen, Shih-Hsin Hsiao, Chi-Li Chung

**Affiliations:** 1 Department of Nursing, MacKay Junior College of Medicine, Nursing, and Management, Taipei 112, Taiwan; s519@mail.mkc.edu.tw; 2 Division of Pulmonary Medicine, Department of Internal Medicine, Taipei Medical University Hospital, Taipei 110, Taiwan; drcarene@tmu.edu.tw; 3 School of Respiratory Therapy, College of Medicine, Taipei Medical University, Taipei 110, Taiwan; 4 Graduate Institute of Clinical Medicine, College of Medicine, Taipei Medical University, Taipei 110, Taiwan; 123013@tmuh.org.tw; 5 Division of Pulmonary Medicine, Department of Internal Medicine, School of Medicine, College of Medicine, Taipei Medical University, Taipei 110, Taiwan

**Keywords:** high mobility group box 1 protein, matrix metalloproteinase-1, mesothelial-to-mesenchymal transition, pleural fibrosis, pleural mesothelial cell, tuberculous pleural effusion, JNK/AP-1 signaling

## Abstract

High-mobility group box 1 (HMGB1) has been implicated in matrix remodeling and fibrotic disorders; however, its role in tuberculosis (TB)-associated pleural fibrosis remains unknown. Pleural fluid levels of HMGB1 and matrix metalloproteinase-1 (MMP-1) in patients with TB pleural effusion (TBPE, *n* = 36) or transudative pleural effusion (TPE, *n* = 14) were measured. The effects of *Mycobacterium tuberculosis* H37Ra (MTBRa) on HMGB1 and MMP-1 expression and their effects on mesothelial–mesenchymal transition (MMT) in human pleural mesothelial cells (PMCs) were assessed. The levels of HMGB1 and MMP-1 were significantly higher in TBPE than in TPE. Elevated HMGB1 and MMP-1 levels in TBPE were positively correlated and both factors were significantly associated with post-TB residual pleural thickening (RPT), particularly in patients with RPT ≥ 10 mm. Colocalized expression of HMGB1 and MMP-1 was also observed in the pleural mesothelium of TBPE patients. MTBRa significantly induced HMGB1 expression in PMCs through activation of the JNK/AP-1 signaling pathway, leading to MMT, enhanced collagen synthesis, and upregulation of MMP-1. Furthermore, silencing of MMP-1 markedly attenuated HMGB1-triggered MMT response. Collectively, HMGB1 promotes pleural fibrogenesis through JNK/AP-1-dependent and MMP-1-mediated MMT, suggesting that targeting the HMGB1/MMP-1 axis may represent a potential therapeutic strategy for TB-related pleural fibrosis.

## 1. Introduction

Tuberculosis (TB) continues to be a significant worldwide public health concern [1]. Tuberculous pleural effusion (TBPE) represents the most prevalent form of extrapulmonary TB and is frequently complicated by residual pleural fibrosis, leading to restrictive pulmonary deficits and symptomatic dyspnea [2]. TBPE results from tuberculous insult to the pleura and is characterized by an accumulation of inflammatory cells, various cytokines, coagulation factors and damage-associated molecular pattern (DAMP) molecules [3]. Growing evidence depicts a correlation between these inflammatory mediators and the severity of inflammation, which contributes to the development of pleural fibrosis [4].

High-mobility group box 1 (HMGB1) is an abundant non-histone nuclear protein involved in the regulation of diverse biological processes. When released extracellularly, HMGB1 functions as an alarmin and a DAMP molecule, alerting the immune system to tissue injury and amplifying inflammatory responses. It exerts its effects through interactions with several receptors, most notably the receptor for advanced glycation end products (RAGE) and Toll-like receptors (TLRs), which activate downstream signaling pathways such as nuclear factor kappa B (NF-κB). This activation promotes the secretion of proinflammatory cytokines and chemokines, thereby recruiting and activating immune cells to sustain inflammation [5]. Moreover, sustained HMGB1 release can initiate fibrotic processes by activating fibroblast-myofibroblast transition (FMT) and inducing matrix metalloproteinases (MMPs), promoting extracellular matrix (ECM) deposition and remodeling. Accordingly, HMGB1 is increasingly recognized as a key mediator and a potential therapeutic target in a broad range of fibrotic diseases [6,7,8,9,10]. However, the expression of HMGB1 in TBPE and its regulatory role in TB pleural fibrosis have never been investigated.

MMPs are zinc-dependent endopeptidases with diverse roles in modulating inflammation and tissue repair and/or remodeling. In the context of tissue repair, they exert dualistic influences, serving as both drivers and suppressors of pathological fibrosis depending on the microenvironment [11]. The levels of MMP-1, also known as collagenase-1, were found to be markedly elevated in patients with TBPE compared to those with heart failure-associated transudates, and were associated with the severity of residual pleural fibrosis [12]. Nevertheless, the precise functional role of MMP-1 in the pathogenesis of TB pleural fibrosis remains to be elucidated.

Our previous research established that TB pleural fibrosis is initiated by *Mycobacterium tuberculosis* (MTB)-induced pleural injury, which activates pleural mesothelial cells (PMCs) and drives their mesothelial–mesenchymal transition (MMT) and extracellular matrix (ECM) production [4,13]. These processes orchestrate the progression toward pleural fibrosis, representing key mechanisms of pleural fibrogenesis. The present study aimed to evaluate the expression profile of HMGB1 in TBPE and investigate its mechanistic interplay with MMP-1 during MTB-activated MMT in human PMCs. Furthermore, we sought to delineate the underlying signaling pathways and the translational significance of these mediators in the development of TB pleural fibrosis.

## 2. Results

### 2.1. HMGB1 and MMP-1 Levels Are Elevated in TBPE and Positively Correlated

**Figure 1:**
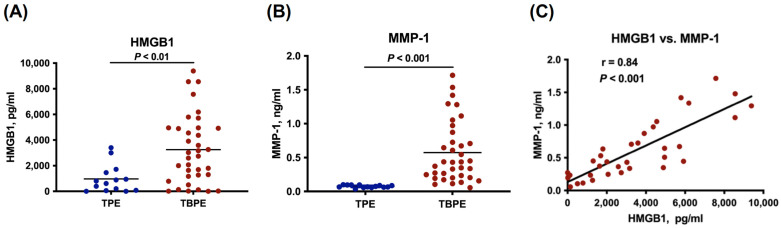
Effusion levels of (**A**) HMGB1 and (**B**) MMP-1 between transudative pleural effusion (TPE, *n* = 14) and tuberculous pleural effusion (TBPE, *n* = 36), and (**C**) the correlation between HMGB1 and MMP-1 in TBPE (*n* = 36). HMGB1, high-mobility group box 1; MMP-1, matrix metalloproteinase-1.

A total of 50 patients were enrolled, including 36 with TBPE and 14 with transudative pleural effusion (TPE). Baseline characteristics are summarized in Table 1. Age, sex, and pleural effusion chest radiograph (CXR) scores were comparable between the two groups. Pleural fluid from patients with TBPE showed significantly higher leukocyte counts, lactate dehydrogenase (LDH), and adenosine deaminase (ADA) levels than that from patients with TPE. After 6 months of anti-TB treatment, 12 of the 36 patients with TBPE developed clinically significant pleural fibrosis, defined as residual pleural thickening (RPT) ≥ 10 mm. To explore the potential profibrotic role of HMGB1 in TBPE, pleural fluid HMGB1 and MMP1 levels were measured. As shown in Figure 1, both HMGB1 and MMP1 levels were significantly elevated in TBPE compared with TPE. In addition, HMGB1 levels were positively correlated with MMP1 levels (*p* < 0.001), suggesting that HMGB1 may participate in the pathogenesis of TBPE through regulation of MMP1.

### 2.2. HMGB1 and MMP-1 Levels in TBPE Positively Correlate with Residual Pleural Fibrosis

**Figure 2:**
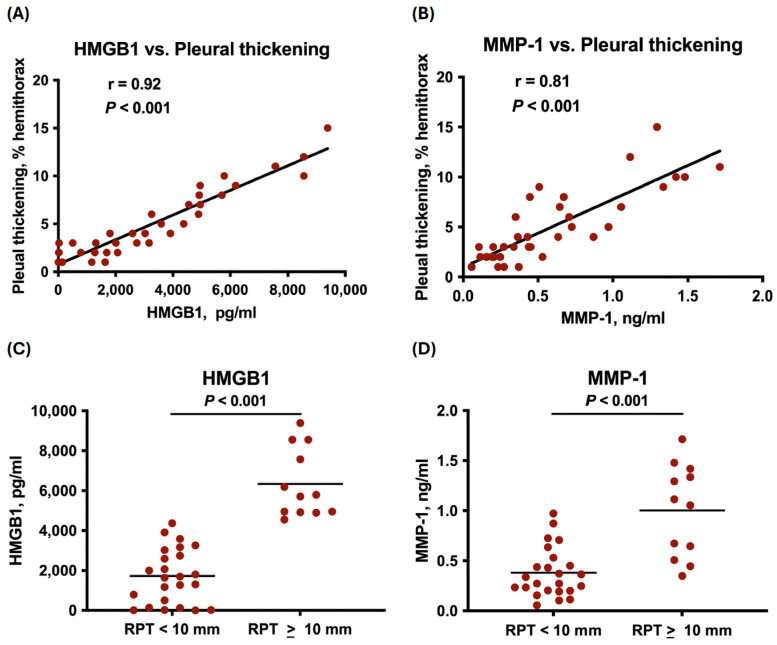
Relationship between effusion levels of HMGB1 or MMP-1 and residual pleural fibrosis in TBPE (*n* = 36). Correlation between effusion levels of (**A**) HMGB1 or (**B**) MMP-1 and residual pleural thickening CXR score (% of hemithorax) in patients with TBPE. Effusion levels of (**C**) HMGB1 and (**D**) MMP-1 in TBPE with RPT ≥ 10 mm (*n* = 12) and TBPE with RPT < 10 mm (*n* = 24); RPT, residual pleural thickening; CXR, chest x-ray.

Pleural fluid HMGB1 and MMP-1 levels were positively correlated with the extent of pleural thickening in patients with TBPE (Figure 2A,B). Furthermore, TBPE patients who had RPT ≥ 10 mm exhibited significantly higher levels of HMGB1 and MMP-1 than those with RPT < 10 mm (Figure 2C,D). These findings suggest that HMGB1 and MMP-1 contribute to the pathogenesis of pleural fibrosis in TBPE.

### 2.3. HMGB1 and MMP-1 Are Expressed in the Pleural Mesothelium of Patients with TBPE

**Figure 3:**
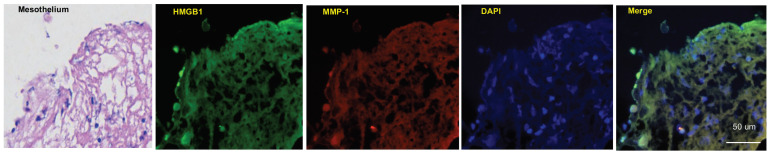
Expression of HMGB1 and MMP-1 in pleural mesothelium from patients with tuberculous pleural effusion (TBPE). Representative H&E staining of subpleural lung tissue sections and immunofluorescence labeling of HMGB1 (green) and MMP-1 (red) in paraffin-embedded pleural mesothelium from TBPE patients. Nuclei were counterstained with DAPI (blue). Scale bar, 50 μm. HMGB1, high-mobility group box 1; MMP-1, matrix metalloproteinase-1; DAPI, 4′,6-diamidino-2-phenylindole.

Hematoxylin and eosin (H&E) staining demonstrated the histological architecture of pleural biopsy specimens from patients with TBPE (Figure 3). Immunofluorescence staining demonstrated HMGB1 and MMP-1 expression in the pleural mesothelial and submesothelial layers. Merged images demonstrated co-localization of HMGB1 and MMP-1 in fibrotic pleural tissue, suggesting their involvement in pleural fibrotic remodeling in TBPE.

### 2.4. MTBRa Upregulates HMGB1 Expression via Activation of JNK Signaling in Human PMCs

**Figure 4:**
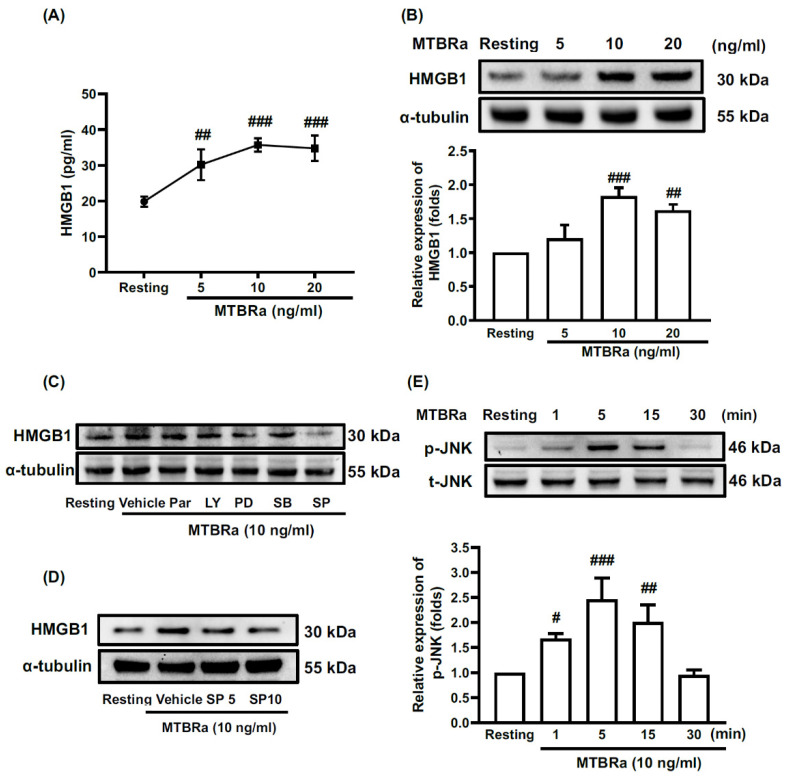
MTBRa upregulates HMGB1 expression via activation of JNK signaling in human PMCs. MeT-5A cells were treated with various concentrations (5–20 ng/mL) of MTBRa for 24 h. (**A**) Levels of secreted HMGB1 in culture supernatant were analyzed by ELISA. (**B**) Intracellular HMGB1 protein expression was analyzed by Western blot. (**C**) Cells were pretreated with vehicle, Parthenolide (Par; NF-κB inhibitor), LY294002 (LY; AKT inhibitor), PD98059 (PD; ERK inhibitor), SB20358 (SB; P38 inhibitor), or SP600125 (SP; JNK inhibitor) prior to MTBRa (10 ng/mL) stimulation for 24 h. (**D**) Cells were pretreated with SP600125 (5 or 10 μM/mL) followed by MTBRa stimulation for 24 h. (**E**) Cells were treated with MTBRa for the indicated time points. HMGB1 expression and JNK phosphorylation were assessed by Western blot using antibodies against total and phosphorylated proteins. Data are presented as mean ± SEM of 3 independent experiments. ^#^ *p* < 0.05, ^##^ *p* < 0.01, ^###^ *p* < 0.001 vs. resting control.

To investigate the mechanism underlying elevated HMGB1 levels in TBPE (Figure 1A), MeT-5A human PMCs were treated with heat-killed Mycobacterium tuberculosis H37Ra (MTBRa) at concentrations of 5, 10, and 20 ng/mL for 24 h. MTBRa stimulation significantly increased HMGB1 secretion (Figure 4A) and intracellular protein expression (Figure 4B) in MeT-5A cells compared with baseline. To identify the signaling pathways involved, pharmacological inhibitors of NF-κB, PI3K/AKT, and MAPK pathways were applied prior to MTBRa stimulation. Among these inhibitors, only the JNK inhibitor SP600125 (5 and 10 μM) significantly attenuated MTBRa-induced HMGB1 expression (Figure 4C,D). Consistently, MTBRa rapidly increased JNK phosphorylation within 5 min (Figure 4E). These findings indicate that MTBRa upregulates HMGB1 expression in PMCs predominantly via JNK pathway activation.

### 2.5. MTBRa Induces c-Jun Activation and AP-1 Transcriptional Activity in Human PMCs

**Figure 5:**
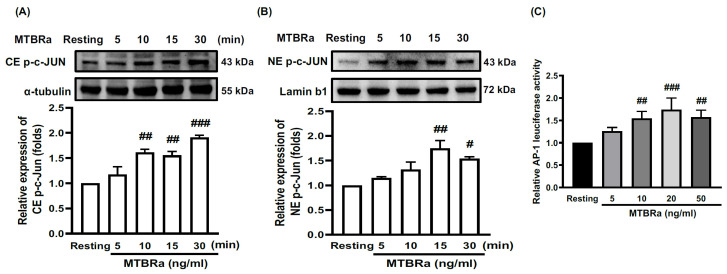
MTBRa induces c-Jun activation and AP-1 transcriptional activity in human PMCs. Cells were pretreated with MTBRa for the indicated times. Cytosolic (**A**) and nuclear (**B**) phosphorylated c-Jun levels were analyzed by Western blot using specific antibodies. (**C**) Cells were cotransfected with an AP-1 luciferase reporter plasmid and a Renilla luciferase vector for normalization of transfection efficiency, followed by MTBRa stimulation for 24 h. AP-1 transcriptional activity was subsequently determined by luciferase assay. Data are presented as mean ± SEM of 3 independent experiments. ^#^ *p* < 0.05, ^##^ *p* < 0.01, ^###^ *p* < 0.001 vs. resting control. CE, cytosolic extract; NE, nuclear extract.

Based on our previous study demonstrating that AP-1 plays a pivotal role in pleural fibrogenesis [13], we investigated whether c-Jun activation and nuclear translocation are required for MTBRa-induced HMGB1 expression in human PMCs. As shown in Figure 5A,B, MTBRa induced c-Jun phosphorylation in the cytoplasm and enhanced its nuclear translocation in human PMCs. Cytoplasmic c-Jun phosphorylation peaked at 30 min, while maximal nuclear accumulation was detected 15–30 min after MTBRa stimulation. Moreover, we evaluated MTBRa-induced AP-1 transcriptional activity in human PMCs using a luciferase reporter assay. MTBRa significantly enhanced AP-1 transcriptional activity at concentrations ranging from 10 to 50 ng/mL (Figure 5C), suggesting that activation of the c-Jun/AP-1 pathway contributes to MTBRa-induced HMGB1 expression.

### 2.6. HMGB1 Induces MMT, Collagen Production, and MMP-1 Expression in Human PMCs

**Figure 6:**
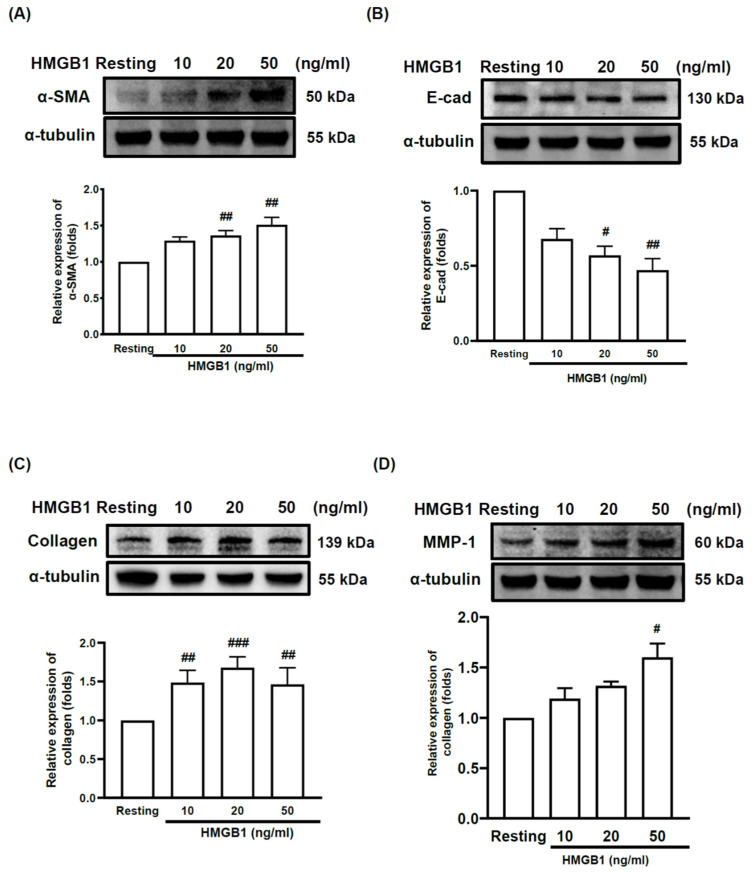
HMGB1 induces MMT, collagen production, and MMP-1 expression in human PMCs. Cells were treated with indicated concentrations of HMGB1 (10, 20, and 50 ng/mL) for 24 h. Protein levels of (**A**) α-SMA, (**B**) E-cadherin (E-cad), (**C**) collagen, and (**D**) MMP-1 were determined by Western blot. Data represent the mean ± SEM of 3 independent experiments. ^#^ *p* < 0.05, ^##^ *p* < 0.01, ^###^ *p* < 0.001 vs. resting control.

Given the elevated levels of HMGB1 and MMP-1 in pleural effusions from TBPE patients, as well as their increased expression in human pleural specimens and PMCs (Figure 3 and Figure 4A,B), we hypothesized that HMGB1 and MMP-1 contribute to fibrotic progression in TBPE. In human PMCs, HMGB1 significantly increased α-SMA expression (Figure 6A) and decreased E-cadherin expression (Figure 6B), consistent with the induction of MMT. Furthermore, HMGB1 enhanced collagen production at concentrations up to 20 ng/mL (Figure 6C) and increased MMP-1 expression at concentrations up to 50 ng/mL (Figure 6D). These findings suggest that HMGB1 promotes pleural fibrotic remodeling by inducing MMT, stimulating collagen synthesis, and enhancing MMP-1-mediated ECM remodeling.

### 2.7. Silencing of HMGB1 Inhibits MTBRa-Mediated Profibrotic Responses in Human PMCs

**Figure 7:**
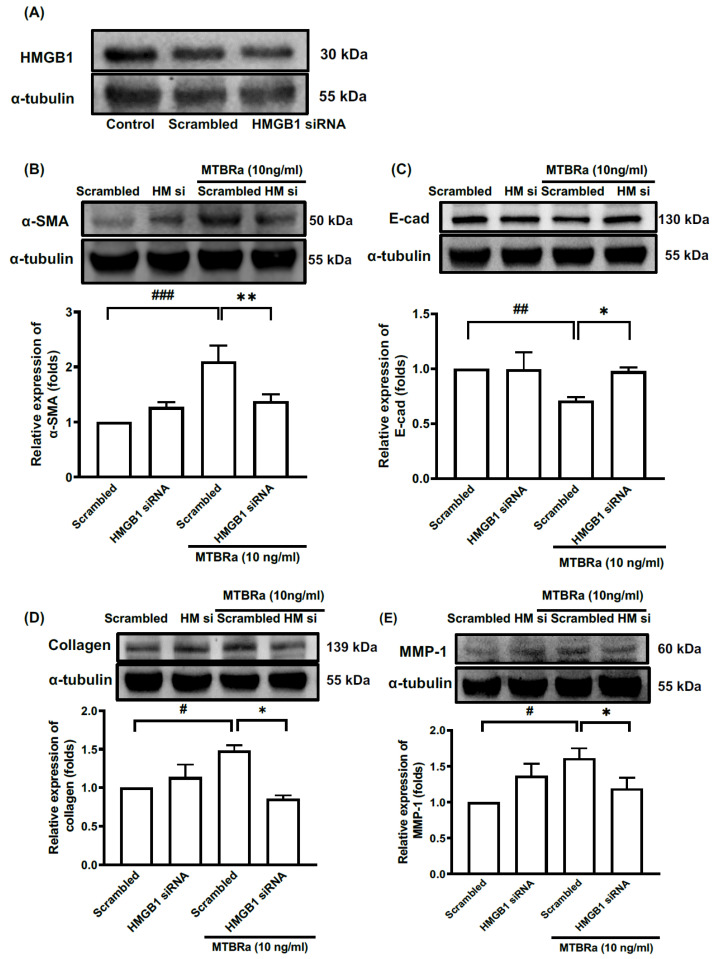
Silencing of HMGB1 inhibits MTBRa-mediated profibrotic responses. MeT-5A cells were transfected with either scramble siRNA (control) or HMGB1-specific siRNA for 24 h, followed by stimulation with MTBRa for an additional 24 h. (**A**) Representative Western blot of HMGB1 to confirm transfection efficiency. Protein levels of (**B**) α-SMA, (**C**) E-cadherin (E-cad), (**D**) collagen, and (**E**) MMP-1 were determined by Western blot. Data are presented as mean ± SEM of three independent experiments. ^#^ *p* < 0.05, ^##^ *p* < 0.01, ^###^ *p* < 0.001 vs. scramble siRNA control; * *p* < 0.05, ** *p* < 0.01, vs. scrambled siRNA + MTBRa group. HMGB1 siRNA, HM si.

We further investigated whether HMGB1 mediates the profibrotic responses in human PMCs following MTBRa infection. We employed a specific siRNA to effectively silence HMGB1 expression (Figure 7A). While MTBRa treatment induced MMT, characterized by upregulated α-SMA and downregulated E-cadherin, these phenotypic changes were significantly attenuated upon HMGB1 knockdown (Figure 7B,C). Furthermore, HMGB1 silencing effectively reversed the MTBRa-mediated induction of collagen and MMP-1 expression (Figure 7D,E). Collectively, these findings demonstrate that HMGB1 plays a pivotal role in the pathogenesis of TB-associated pleural fibrosis. By driving MMT, stimulating excessive collagen production, and upregulating MMP-1, HMGB1 acts as a central mediator of tissue remodeling. Consequently, our data identify HMGB1 as a promising therapeutic target for the prevention of pleural fibrosis in patients with TBPE.

### 2.8. MMP-1 Mediates HMGB1-Induced MMT in Human PMCs

**Figure 8:**
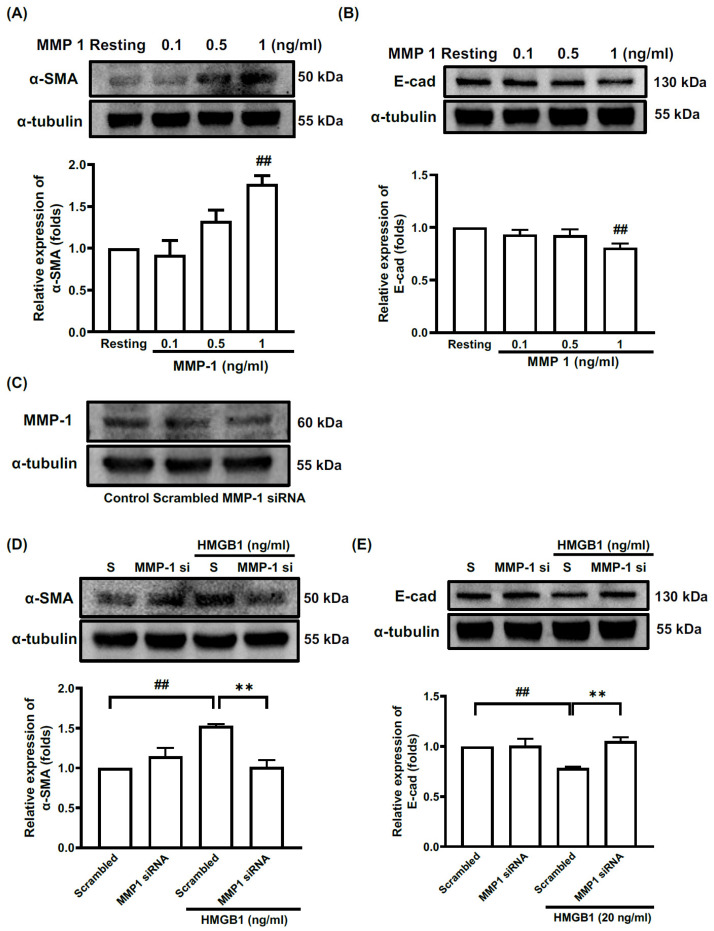
MMP-1 mediates HMGB1-induced MMT in human PMCs. MeT-5A cells were treated with various concentrations of active recombinant MMP-1 (0.1, 0.5, and 1 ng/mL) for 24 h. Protein expression levels of (**A**) α-SMA and (**B**) E-cadherin (E-cad) were then assessed. (**C**) Knockdown efficiency of MMP-1 siRNA was confirmed. MeT-5A cells were transfected with either scrambled siRNA (control) or MMP-1-specific siRNA for 24 h, followed by stimulation with HMGB1 for an additional 24 h. Following MMP-1 silencing, the expression levels of (**D**) α-SMA and (**E**) E-cadherin were further evaluated. All protein expressions were analyzed by Western blot. Data are presented as mean ± SEM of three independent experiments. ^##^ *p* < 0.01 vs. scrambled siRNA control; ** *p* < 0.01 vs. scrambled siRNA + HMGB1 group. Scrambled siRNA, S; MMP-1 siRNA, MMP-1 si.

Based on the positive correlation between HMGB1 and MMP-1 levels in TBPE (Figure 1C), and the previous report indicating that MMP-1 knockdown inhibits the epithelial–mesenchymal transition (EMT) via PI3K/Akt/c-Myc signaling [14], we sought to determine whether MMP-1 is a requisite mediator of HMGB1-induced MMT in human PMCs. Direct stimulation of PMCs with active recombinant MMP-1 (0.1, 0.5, 1 ng/mL) significantly upregulated α-SMA expression while concurrently downregulating E-cadherin (Figure 8A,B). Transfection with specific MMP-1 siRNA markedly reduced MMP-1 protein expression, confirming the knockdown efficiency (Figure 8C). Furthermore, MMP-1 silencing significantly attenuated HMGB1-induced phenotypic transitions, as characterized by a suppressed upregulation of α-SMA (Figure 8D) and a restoration of E-cadherin expression (Figure 8E). These results identify MMP-1 as an essential downstream mediator of HMGB1 signaling, and indicate that the HMGB1/MMP-1 axis drives MMT and fibrotic remodeling in human PMCs.

Collectively, these findings indicate that *M. tuberculosis* activates JNK/AP-1 signaling in PMCs, leading to HMGB1 upregulation. Elevated HMGB1 subsequently induces MMP-1 expression and MMT, thereby promoting ECM production and remodeling and contributing to pleural thickening and tuberculous pleural fibrosis (Graphical abstract).

## 3. Discussion

The present study demonstrated that pleural fluid levels of HMGB1 and MMP-1 were significantly elevated in TBPE, and were positively correlated and associated with post-TB pleural fibrosis. Furthermore, MTBRa induced HMGB1 expression through activation of the JNK/AP-1 signaling pathway, leading to MMT, collagen production, and MMP-1 upregulation. Silencing of MMP-1 markedly attenuated HMGB1-induced MMT. To our knowledge, this is the first study to demonstrate the clinical significance of HMGB1 and its profibrotic effects in TBPE, including HMGB1-mediated MMT and ECM production through JNK/AP-1 signaling and MMP-1-dependent mechanisms.

Extracellular HMGB1 can promote inflammatory processes or EMT, facilitating the migration and activation of mesenchymal cells and contributing to ECM deposition and tissue fibrosis [6]. Comparably, MMP-1 plays a multifaceted role in fibrogenesis by modulating ECM turnover, inflammation, and myofibroblast transformation [12,14,15]. However, the fibrogenic effect of HMGB1 and MMP-1 in TBPE and their impact on clinical outcome have never been investigated. A previous study demonstrated elevated HMGB1 expression in malignant and inflammatory pleural and peritoneal effusions [16], while our prior work showed that pleural fluid MMP-1 levels correlate with residual pleural fibrosis in TBPE [12]. In the present study, we found for the first time that HMGB1 levels were significantly increased in TBPE and were strongly associated with MMP-1 levels. Consistently, MTBRa markedly induced HMGB1 expression in human PMCs, supporting a mechanistic link between clinical and in vitro findings. Clinically, HMGB1 and MMP-1 levels were significantly higher in TBPE patients with RPT ≥ 10 mm and positively correlated with residual pleural opacity on chest radiography. Histopathological and immunofluorescence analyses further demonstrated extensive pleural fibrosis and elevated HMGB1 and MMP-1 expression in mesothelial and fibrotic regions, suggesting that both mediators contribute to fibrotic remodeling in TBPE.

Given that MTB infection elicits inflammatory responses through activation of multiple intracellular signaling pathways [17], we next investigated the specific pathways responsible for MTB-induced HMGB1 production in human PMCs. Pretreatment with the JNK inhibitor significantly attenuated MTBRa-induced HMGB1 production. Correspondingly, MTBRa stimulated JNK phosphorylation in human PMCs, promoting the cytoplasmic accumulation and subsequent nuclear translocation of c-Jun, which in turn enhanced AP-1 promoter activity. In agreement with reported mechanisms in macrophages [18], the present findings demonstrated that MTB induces HMGB1 expression primarily through the JNK/AP-1 signaling axis. This specific pathway appears to be the central regulator of the inflammatory activation of PMCs in response to MTB infection [19].

HMGB1 functions as an extracellular DAMP during tissue injury to trigger pro-inflammatory cytokine secretion and sustain immune cell recruitment [5]. Beyond driving inflammation, chronic HMGB1 release promotes pathological fibrogenesis by stimulating the FMT and inducing MMPs to accelerate ECM deposition and remodeling [20]. Prior studies revealed that pleural injury serves as the primary promoter for TB-associated fibrosis by inducing mesothelial cell activation, MMT and ECM deposition [21]. While PMCs are known to undergo MMT in response to thrombin [4], our study demonstrated a similar role for HMGB1 in TB pleurisy. Specifically, HMGB1 induced MMT, and concurrently stimulated collagen and MMP-1 production. Notably, HMGB1 silencing effectively abrogated MTBRa-induced profibrotic responses, suggesting that MTB drives MMT and ECM deposition substantially through an HMGB1-mediated mechanism.

Moreover, because HMGB1 upregulates MMP-1 and both correlate with pleural fibrosis in TBPE, elucidating their mechanistic interplay in pleural fibrogenesis is highly warranted. Clinical evidence links MMP-1 polymorphisms to severe post-tuberculosis tracheobronchial stenosis and pulmonary fibrosis [22,23]. Similarly, MMP-1 is markedly upregulated in idiopathic pulmonary fibrosis (IPF) tissue, though its pro-fibrotic mechanisms have remained elusive [24]. Classically, MMP-1 is recognized as an interstitial collagenase that mediates ECM degradation [25]; however, the biological functions of MMP-1 extend beyond simple proteolysis. For example, MMP-1 has been shown to promote EMT in various cancer types [14,26], mechanistically modulating cellular phenotypic transitions via protease-activated receptor-1 signaling [26]. Furthermore, in fibrotic pathologies such as renal fibrosis, MMP-1, like other MMPs, may drive basement membrane degradation, E-cadherin disruption, and latent transforming growth factor-β (TGF-β) activation to induce EMT [27,28,29]. Collectively, these processes demonstrate that MMP-1 functions not merely as a collagenolytic enzyme, but as a central orchestrator of fibrotic remodeling. In parallel, our findings revealed that during MTB stimulation, the capacity of MMP-1 to drive MMT outpaces its classical collagenolytic activity. Specifically, MMP-1 significantly promoted MMT, whereas its silencing reversed HMGB1-induced MMT, indicating that MMP-1 acts as a key downstream effector involved in HMGB1-mediated fibrotic cascades. Together, these results highlight the HMGB1/MMP-1 axis as a pivotal pathway orchestrating mesothelial transition and tissue remodeling in TB pleural fibrogenesis.

Several limitations of this study should be acknowledged. First, although HMGB1 and MMP-1 levels correlate significantly with post-TB pleural fibrosis, their direct impact on long-term clinical outcomes remains to be fully defined. Larger prospective cohorts are warranted to validate whether effusion HMGB1 or MMP-1 can serve as reliable predictive or prognostic biomarkers in TBPE. Second, while we demonstrated that the HMGB1/MMP-1 axis mediates MMT and profibrotic pathways in vitro, the therapeutic efficacy of targeting this axis has yet to be explored in vivo. Despite these limitations, to our knowledge, this is the first study to characterize both the cellular mechanisms and clinical relevance of the HMGB1/MMP-1 pathway in TBPE, providing a novel framework for future targeted therapeutics against pleural fibrosis.

In conclusion, this study establishes HMGB1 as a key mediator of mesothelial activation and fibrogenesis in TBPE via JNK/AP-1 and MMP-1 signaling. By linking clinical data to cellular mechanisms, we provide novel insights into TB pleural fibrosis and propose the HMGB1/MMP-1 axis as a therapeutic target to mitigate inflammation-driven remodeling. Further clinical validation remains essential to translate these findings into effective anti-fibrotic treatments.

## 4. Materials and Methods

### 4.1. Materials

Heat-killed *Mycobacterium tuberculosis* H37Ra and specific inhibitors were purchased from Sigma-Aldrich (St. Louis, MO, USA) and Calbiochem (San Diego, CA, USA), respectively. Recombinant HMGB1 was obtained from BioLegend (San Diego, CA, USA). Primary antibodies were sourced from GeneTex (Irvine, CA, USA), while the secondary anti-rabbit IgG antibody was purchased from Jackson ImmunoResearch (West Grove, PA, USA).

### 4.2. Patient Enrolment

Patients with pleural effusions of unknown etiology admitted to Taipei Medical University Hospital were consecutively enrolled and categorized into two groups: TBPE (*n* = 36) and TPE (*n* = 14). All patients underwent pleural fluid acid-fast bacilli staining and mycobacterial culture, with a pleural biopsy additionally performed when TBPE was clinically suspected. A definitive diagnosis of TBPE was established by the detection of MTB in pleural fluid or tissue, or by the presence of caseating granulomas in pleural biopsy specimens, regardless of acid-fast bacilli staining results. All TBPE patients received standard anti-tuberculosis treatment following diagnosis.

### 4.3. Pleural Fluid and CXR Analysis

Within 24 h of admission, thoracentesis was performed by a thoracic specialist to collect 50 mL of pleural effusion for routine fluid analysis and microbiological testing. Posteroanterior CXRs were obtained at baseline and every two months thereafter for a minimum of six months. To evaluate the extent of lateral pleural thickening, two independent radiologists, blinded to the clinical data, reviewed the images via the Picture Archiving and Communication System (PACS). Residual pleural thickening (RPT), defined as a maximum linear width of the pleural opacity, and RPT ≥ 10 mm was used as the indicator for clinically significant pleural fibrosis. All procedures were conducted following the receipt of written informed consent from all participants. This study was approved by the Institutional Review Board of Taipei Medical University Hospital, Taiwan (IRB No. N202505002).

### 4.4. Tissue Immunofluorescence and H&E Staining

Paraffin-embedded parietal pleural biopsies from TBPE patients were sectioned and mounted onto glass slides. The sections were deparaffinized in xylene, rehydrated through a series of graded ethanol solutions, and washed twice with PBS. For immunofluorescence staining, the slides were incubated with primary antibodies against HMGB1 or MMP-1, followed by incubation with FITC- or Cy5-conjugated secondary antibodies. Nuclei were counterstained with 4′,6-diamidino-2-phenylindole (DAPI), and the sections were mounted using an anti-fade mounting medium. Images were captured using an Olympus microscope imaging system with OlyVIA software (version 3.2.1; Olympus, Tokyo, Japan). Additionally, consecutive sections were stained with hematoxylin and eosin (H&E) to evaluate tissue morphology and pathological features. These histological images were acquired using a digital slide scanner equipped with Mitotic Digital Slide Assistant software (version 1.0.7.46; Mitotic Asia, Kowloon, Hong Kong).

### 4.5. Cell Culture

The MeT-5A human pleural mesothelial cell line (ATCC #CRL-9444) was obtained from the American Type Culture Collection (Manassas, VA, USA) and cultured as previously described [13].

### 4.6. Enzyme-Linked Immunosorbent Assay (ELISA)

Pleural fluid samples from patients with TBPE and TPE were collected as previously described [4]. The concentrations of HMGB1 and MMP-1 in the effusions were determined using commercial ELISA kits (R&D Systems, Minneapolis, MN, USA) according to the manufacturer’s instructions. Furthermore, HMGB1 levels in the culture supernatants of MeT-5A cells following stimulation with MTBRa were similarly quantified by ELISA.

### 4.7. Western Blot

Proteins were separated via 10% SDS-PAGE and electrotransferred onto nitrocellulose membranes (Millipore, Billerica, MA, USA). After blocking with 5% non-fat milk, the membranes were incubated overnight at 4 °C with specific primary antibodies against the indicated proteins. Subsequently, the membranes were incubated with horseradish peroxidase (HRP)-conjugated secondary antibodies. Protein bands were visualized using enhanced chemiluminescence reagents, and quantitative densitometric analysis was performed using the ChemiDoc MP Imaging System (Bio-Rad, Hercules, CA, USA). The intensity of each target protein was normalized to its corresponding loading control, and the normalized values were expressed relative to the control group.

### 4.8. Preparation of Nuclear–Cytoplasmic Proteins

MeT-5A cells were seeded at a density of 6 × 10^5^ cells in 10 cm dishes and cultured for 48 h. Following serum starvation in serum-free medium for 24 h, the cells were exposed to MTBRa for the indicated time periods. Nuclear and cytoplasmic proteins were then isolated using the NE-PER™ Nuclear and Cytoplasmic Extraction Reagents (Pierce, Rockford, IL, USA) according to the manufacturer’s instructions. α-tubulin was used as the loading control for whole-cell lysates, whereas lamin B1 was used as the loading control for nuclear protein extracts.

### 4.9. RNA Interference

MeT-5A cells were transfected with small interfering RNA (siRNA) targeting HMGB1, MMP1, or scrambled (non-targeting) siRNA using the DharmaFECT^®^ Transfection Reagent (Thermo Scientific, Waltham, MA, USA) according to the manufacturer’s instructions. The siRNA complexes were added to the cells in antibiotic-free M199 medium for 24 h. Subsequently, the medium was replaced with serum-free medium for another 24 h to induce starvation prior to treatment with MTBRa, recombinant HMGB1, or recombinant MMP-1. The efficiency of protein knockdown and subsequent expression changes were analyzed by Western blotting.

### 4.10. Statistical Analyses

Statistical analyses were performed using GraphPad Prism software (version 9.3.1; GraphPad Software, San Diego, CA, USA). Comparisons between two groups were conducted using the unpaired Student’s *t*-test or the Mann–Whitney U test, depending on data distribution. For comparisons among three or more groups in the Western blot experiments, one-way analysis of variance (ANOVA), followed by the Student–Newman–Keuls (SNK) post hoc test, was employed. Spearman’s rank correlation was used to evaluate the relationships between continuous variables. Categorical variables were analyzed using the chi-square (χ^2^) test or Fisher’s exact test, as appropriate. Clinical data were expressed as median (range), whereas Western blot experimental data were expressed as the mean ± standard error of the mean (SEM). A *p*-value < 0.05 was considered statistically significant.

## Figures and Tables

**Table 1:** Demographics and pleural fluid characteristics *.

	TPE	TBPE	*p*-Value
Subject, *n*	14	36	
Age, years	82 (56–92)	76 (25–90)	0.061
Males, *n* (%)	7 (50)	28 (78)	0.085
Pleural effusion CXR score, %	58 (47–63)	43 (21–56)	0.213
Pleural fluid			
pH value	7.38 (7.33–7.45)	7.33 (6.92–7.53)	0.063
Glucose, mg/dL	110 (84–167)	118 (24–285)	0.583
LDH, IU/dL	112 (48–121)	286 (78–2032)	<0.001
Leukocyte count, cells/μL	268 (123–382)	1158 (88–16,792)	<0.01
ADA, IU/L	25 (7–36)	115 (56–243)	<0.0001

Abbreviations: TPE, transudative pleural effusion; TBPE, tuberculous pleural effusion; CXR, chest radiograph; LDH, lactate dehydrogenase; ADA, adenosine deaminase. * Values are expressed as median (range) unless otherwise specified.

## Data Availability

The original contributions presented in this study are included in the article. Further inquiries can be directed to the corresponding author.

## Abbreviations

The following abbreviations are used in this manuscript:

HMGB1	High-mobility group box 1
MMP-1	Matrix metalloproteinase-1
MMT	Mesothelial–mesenchymal transition
PMC	Pleural mesothelial cell
RPT	Residual pleural thickening
ECM	Extracellular matrix
TBPE	Tuberculous pleural effusion
